# Components and Anti-HepG2 Activity Comparison of *Lycopodium* Alkaloids from Four Geographic Origins

**DOI:** 10.1155/2016/4631843

**Published:** 2016-02-28

**Authors:** Yong-Qiang Tian, Guang-Wan Hu, Ming-Quan Guo

**Affiliations:** ^1^Key Laboratory of Plant Germplasm Enhancement and Specialty Agriculture, Wuhan Botanical Garden, Chinese Academy of Sciences, Wuhan 430074, China; ^2^Graduate University of Chinese Academy of Sciences, Beijing 100049, China; ^3^Sino-Africa Joint Research Center, Chinese Academy of Sciences, Wuhan 430074, China

## Abstract

*Lycopodium japonicum *Thunb. has attracted great interests due to its rich alkaloids with significant anticancer activity. However, significant chemical differences often exist in a plant species from different geographic origins and affect its quality and bioactivities. Thus, it is urgent to reveal their chemical and biological distinctions at the molecular level. In this context, a comparative chemical analysis of LAs using HPLC-UV-ESI-MS/MS was firstly conducted and resulted in the detection of 46 LAs, 28 of which were identified, and a series of unique LAs markers, such as peaks** 2**,** 9**,** 10**, and** 11**, were further found to be characteristic LAs and selected as markers from four different origins for their quality control. In parallel, the comparative bioactivity assay revealed that the total LAs from Hubei province exhibited much higher inhibitory rate at 65.95% against HepG2 cells than those at 26.72%, 20.26%, and 33.62% for Kenya, Guangxi province, and Zhejiang province in China, respectively. To this end, significant chemical fingerprinting differences and discrepancies in bioactivity of LAs were explored firstly, which could provide valuable information for quality control and further activity studies on LAs from different sources and promote their better pharmaceutical applications in the future as well.

## 1. Introduction

*Lycopodium japonicum* Thunb is a traditional medicinal herb in China, which has been used for the treatment of a variety of diseases for thousands of years, such as contusion, analgesia, and rheumatoid arthritis [1, 2]. It has been reported that lycopodium alkaloids (LAs) were the major bioactive components widely found in the plants of* Lycopodium* genus, for example,* L. obscurum* [3],* L. annotinum* [4], and* L. chinense* [5]. Since these LAs have been proved to possess a wide spectrum of bioactivities, for example, anti-inflammation, antitumor, and acetylcholineasterase inhibitory activity [6–9], many efforts focusing on the isolation, synthesis, identification, and biogenetic synthesis of LAs have been made to explore and expand this valuable medicinal resource [10]. To date, up to 300 LAs were reported mainly from various* Lycopodium* and* Huperzia* genus plants or from one plant with different growing stages and environments [11]. Among them, geoorigin of a plant is an important factor affecting the types and chemical structures of LAs since they are produced through plant metabolism and its complex interactions with the growing environments. Therefore, comparative study on the LAs of Lycopodium genus plants from different areas can provide valuable information on the evaluation of their chemical similarities and differences and further their pharmaceutical activity discrepancies.

To investigate LAs from a plant species, the traditional phytochemical approaches usually involved multistep isolation and structural elucidation of pure compounds from a plant of interest, which are often tedious, complex, and time-consuming [3]. Because of the excellent performance on simultaneous separation and identification of multicomponent mixtures with complex background, chromatography based separation techniques (i.e., GC and LC) coupled with various detectors, such as mass spectrometry (MS), were developed as useful tools in most cases [12]. Since most LAs from plants are nonvolatile, LC techniques, for example, high-performance LC (HPLC) and ultraperformance LC (UPLC), were preferred over GC in obtaining the fingerprint profiles of LAs [13]. However, the UV detector widely used with LC often failed to provide chemical structure information when the authentic standards of LAs were unavailable. Thus, LC-MS was used in this work for the identification of LAs of* Lycopodium japonicum* Thunb from different geoorigins. It is reported that the types and contents of LAs did not only differ in different plant species but also differ in the same plant species originated from different places [14, 15]. It is reported that* Lycopodium japonicum* Thunb is widely distributed in China in Flora of China, which was close but different from* Lycopodium clavatum* [16]. However, lycopodium plants with different growing environments may have significant differences in LAs compositions, and very few efforts have been made to illustrate these differences. Most of the existing study focused on the chemical composition and bioactivity of one or several components of LAs in* Lycopodium* plant species from one place [2], which could pose high risk in practical medical applications or evaluations on the* Lycopodium* plant due to the inconsistent responses of LAs when a* lycopodium* plant species from different places were utilized. Thus, a comparative investigation on the chemical composition and the corresponding bioactivity of LAs in a Lycopodium species from different places will be of special interest in exploring and expanding these natural resources for human health and well-being.

In this work, the fingerprinting profiles of LAs in* Lycopodium japonicum* Thunb from four geoorigins, that is, Hubei, Guangxi, and Zhejiang province of China and Kenya, were firstly determined and compared using high performance liquid chromatography coupled with electrospray ionization mass spectrometry (HPLC/ESI-MS/MS), and the anti-HepG_2_ activities of the corresponding total LAs were also tested* in vitro* with Cell Counting Kit-8 (CCK-8). In this way, the correlations between total LAs and their associated activities were firstly explored, which could provide valuable information for quality control and further activity studies on LAs from different natural resources and could thus promote their better applications in the future.

## 2. Materials and Methods

### 2.1. Plant Materials

Crude* Lycopodium* plants of three geo-origins (Hubei, Guangxi and Zhejiang) were obtained from Xinhui Pharmaceutical Factory (Anhui, China). The Kenya origin* Lycopodium* material was collected from Mountain Kenya, Kenya. The plant materials of the four origins were identified as* Lycopodium japonicum* Thunb of the same species by Guangwan Hu, who is an expert of taxonomy from Wuhan Botanical Garden of Chinese Academy of Sciences. The herb was dried at a temperature of 40°C and pulverized for further experiment.

### 2.2. Chemicals and Reagents

Ammonium acetate (AA) and acetonitrile (ACN) were purchased from ROE Scientific Inc, and Fisher Scientific, respectively. Other solvents, such as methanol, ethanol, chloroform, hydrochloric acid (HCl), and ammonia, were purchased from Sino-pharm chemical Reagent Co., Ltd. (Shanghai, China). Deionized water for HPLC and LC-MS was prepared from EPED (Nanjing Yeap Esselte Technology Development Co., Nanjing, China).

### 2.3. Alkaloids Extraction

For Hubei origin* Lycopodium japonicum*, the pulverized powder (40.0 g) was immersed in 90% ethanol for 12 h and sonically extracted for 30 min three times. After filtration, the filtrate was combined and then concentrated at 45°C with a vacuum rotatory evaporator. The residue was dissolved in 0.5% HCl and then extracted with chloroform three times. The remaining aqueous phase was adjusted to pH 10 using NH_4_OH and further extracted with chloroform to afford the crude alkaloids in this study. In the same way, the total LAs in* Lycopodium japonicum* from Guangxi, Zhejiang province, and Kenya were prepared.

### 2.4. Instrumentation and Chromatographic Conditions

#### 2.4.1. High Performance Liquid Chromatography

A Thermo Accela 1250 HPLC system (Thermo Fisher Scientific, San Jose, CA, USA) equipped with a vacuum degasser, an autosampler, and a variable-wavelength detector (VWD) was used for the HPLC-UV analysis. The separation was carried out on a Waters Xbridge Sunfire C18 (4.6 × 150 mm, 3.5 *μ*m, Waters Technology, Ireland, UK). The column temperature was maintained at 30°C and the flow rate was set at 0.6 mL/min. The mobile phase consisted of 10 mM ammonium acetate aqueous solution (A) and acetonitrile (B). The gradient elution profiles were as follows: 0–3 min, 90% (B); 3–35 min, 90%–50% (B); 35–37 min, 50%–10% (B); 37–40 min, 10% (B). The LC chromatogram was monitored at a wavelength of 279 nm.

#### 2.4.2. Mass Spectrometry

For the LC-ESI-MS/MS experiment, a Thermo Accela 600 HPLC system with a UV detector coupled with a TSQ Quantum Access MAX mass spectrometer (Thermo Fisher Scientific, San Jose, CA, USA) was used. LC-MS analyses were conducted in the positive mode. MS conditions were as follows: mass range from 200 to 1000, spray voltage, 3.0 kV; capillary temperature, 250°C; sheath gas pressure, 40 psi; aux gar pressure, 10 psi.

### 2.5. Anti-HepG2 Activity In Vitro

The anti-HepG2 activity of the crude extracts was tested using human hepatic carcinoma cell line (HepG2, from China Center For Type Culture Collection) with Cell Counting Kit-8 (CCK-8). Cells were cultured in a 96-well plate at a density of 5000 cells per well in DMEM (Dulbecco's 24 Modified Eagle Medium) supplemented with 10% fetal bovine serum (FBS). After culturing under 5% CO_2_ at 37°C for 24 h, the cells were treated with LAs at the concentrations of 10 *μ*g/mL with 3 duplicates. DMSO was used as blank control. After incubation for 48 h, 10 *μ*L of CCK-8 was added to each well. Another 2 hours later, the optical density (OD) values were determined at 450 nm by microliter plate reader (MIOS Junior, Merck). The inhibitory rate (%) = (ODC − ODT)/ODC × 100% [15, 17, 18], where ODT and ODC were the OD values of blank control and LAs, respectively. The triplicate OD values were used to calculate RSDs.

## 3. Results and Discussion

### 3.1. HPLC Fingerprints of LAs in* Lycopodium* from Four Geoorigins

**Figure 1:**
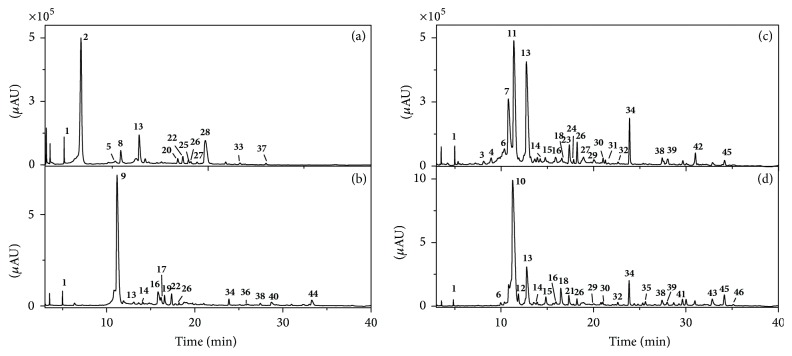
HPLC-UV fingerprints of LAs from four origins: Hubei (a), Kenya (b), Guangxi (c), and Zhejiang (d).

The LAs in* Lycopodium* from four geoorigins were extracted and analyzed by HPLC-UV under the given conditions. Figures 1(a), 1(b), 1(c), and 1(d) show the fingerprint profiles of the LAs corresponding to the four geoorigins. It was observed that the LAs in the four samples were tentatively separated under the given operation conditions. As a result, 46 LAs were detected in this study, which provided valuable chemical information on LAs in* Lycopodium* from different origins.

### 3.2. Identification of Alkaloids

**Figure 2:**
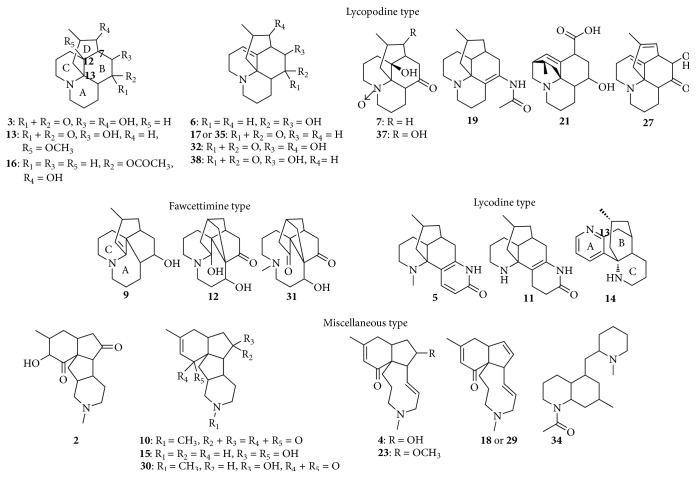
Structures of the LAs identified.

To identify the peaks detected in Figure 1, in-house database of LAs was established based on the investigation of the reported literatures. The information of the 46 detected LAs was summarized in Table 1. Since the diversity and complexity of LAs may result in confusion and difficulty in identifying these alkaloids, the LAs were classified into four groups based on their structures in order to simplify the MS/MS spectrum interpretation, including lycopodine type, lycodine type, fawcettimine type, and miscellaneous type. By comparing with the MS/MS spectrum of authentic standards and the reported fragment pathway, 28 LAs were identified and the structures were shown in Figure 2. The literature investigation of* Lycopodium japonicum* plant species indicated that ten LAs (labeled as “^Δ^” in Table 1) might be identified for the first time in this species. The detailed MS interpretations were elucidated as follows.

#### 3.2.1. Lycopodine Type Alkaloids

With unique structures, the lycopodine type is the largest group among the known LAs [11]. The MS/MS spectra of this type usually exhibited the neutral loss of C_4_H_8_ (56 Da) resulting from the cleavage of D ring in the EI mode, which led to the characteristic cleavage between C-7 and C-13 [19]. However in the positive electrospray ion mode (ESI), the MS/MS spectrum indicated the easier loss of substituents at C-12 [20]. It can thus be deduced that the most abundant fragment ion of lycopodine type alkaloids in the ESI-MS was produced through the loss of substituents at C-12, especially those substituents such as OH or OCH_3_ at C-12, and only in a few cases, the neutral loss of 56 Da (C_4_H_8_) could be observed.

In this study, 12 peaks (peaks** 3**,** 6**,** 7**,** 13**,** 16**,** 17**,** 19**,** 21**,** 27**,** 32**,** 35**,** 37,** and** 38**) were identified and classified into lycopodine type alkaloids. Peaks** 3** and** 7** had the same [M + H]^+^ ion at *m*/*z* 280 and the very similar fragment ions at *m*/*z* 244, 216, and 188. The fragment ion at *m*/*z* 188 was elucidated as the further loss of C_4_H_8_ (56 Da) from the abundant fragment at *m*/*z* 244, indicating the two chemicals belonging to lycopodine type. The distinguished fragments at *m*/*z* 218 were observed in the MS/MS spectrum of peak** 7**, which indicated the neutral loss of O. (16 Da) from fragment at *m*/*z* 234. Thus, peaks** 3** and** 7** were identified as 6*α*,8*β*-dihydroxylycopodine and obscurumine A, respectively, [11, 21]. For peak** 38**, the same fragments at *m*/*z* 244, 234, and 216 as peak** 3** were observed, which indicated their similarity of structures. Peak** 38** showed the [M + H]^+^ ion at *m*/*z* 262 (C_16_H_23_NO_2_) and was identified as lycoposerramine K [22].

Peak** 13** showed the [M + H]^+^ ion at *m*/*z* 294 and yielded abundant fragment ion at *m*/*z* 262 due to the neutral loss of substituent CH_3_OH (32 Da). In addition to the fragments at *m*/*z* 276 [M + H-H_2_O]^+^, 244 [M + H-CH_3_OH-H_2_O]^+^, and 215 [M + H-CH_3_OH-H_2_O-CH_2_NH]^+^, peak** 13** was tentatively identified as 12-methoxyl-lycoposerramine K. For peak** 16**, the most abundant fragment at *m*/*z* 248 indicated the neutral loss of 60 Da (CH_3_COOH). Because of the neutral loss of H_2_O (18 Da), the fragment ion at *m*/*z* 230 was observed. Thus, peak** 16** was identified as *α*-lofoline [11].

With the same [M + H]^+^ at *m*/*z* 278, peaks** 21** and** 32** also exhibited the same fragments at *m*/*z* 260, 242, and 232. In the MS/MS spectrum of peak** 21**, fragments at *m*/*z* 234 and 216 were produced by the loss of CO_2_ (44 Da) and successive loss of H_2_O (18 Da), while the distinguished fragment at *m*/*z* 214 was probably produced by the loss of CO (28 Da) from fragment at *m*/*z* 242. Therefore, peaks** 21** and** 32** were identified as lannotinidine J and 8*β*-lycoposerramine K, respectively, [11, 23]. Although different retention times of peak** 17** and** 35** were observed at 16.16 min and 24.41 min, respectively, they had the same MS/MS spectrum, and were identified as anhydrolycodoline or its stereoisomer [4].

By comparing the MS/MS data with those reported in literatures, peak** 19** had a [M + H]^+^ ion at *m*/*z* 289 and was identified as 5-N-acetyl-4,5-dehydrolycopodine [20]. In its MS/MS spectrum, abundant fragments at *m*/*z* 247 and 230 were produced by the loss of C_2_H_2_O (42 Da) and CH_3_CONH_2_ (59 Da). For peak** 6**, the fragments at *m*/*z* 246 [M + H-H_2_O]^+^, 228 [M + H-2H_2_O]^+^, 218 [M + H-H_2_O-CO]^+^, and 200 [M + H-2H_2_O-C_2_H_4_]^+^ were attributed to the loss of substituents and the subsequent RDA cleavage. Thus, peak** 6** was identified as lucidioline or its stereoisomers [24]. As to peak** 27**, the loss of OH and carbonyl at C-5 yielded fragments at *m*/*z* 242, 232, and 214. Thus, the peak was tentatively identified as 8-alkenyl-lycoposerramine K. For peak** 37**, the successive loss of H_2_O from C-8 and C-12 led to the fragments at *m*/*z* 278 and 260. The abundant fragment at *m*/*z* 234 was produced by the further loss of O and CO. Thus, peak** 37** was identified as miyoshianine C [24].

#### 3.2.2. Lycodine Type Alkaloids

Different from lycopodine type alkaloids with a single nitrogen atom, lycodine type LAs possessed two nitrogen atoms [4]. Thus, in the MS/MS spectra, LAs of this type often showed the most abundant fragments ions due to the loss of N and the vicinal moieties.

**Figure 3:**
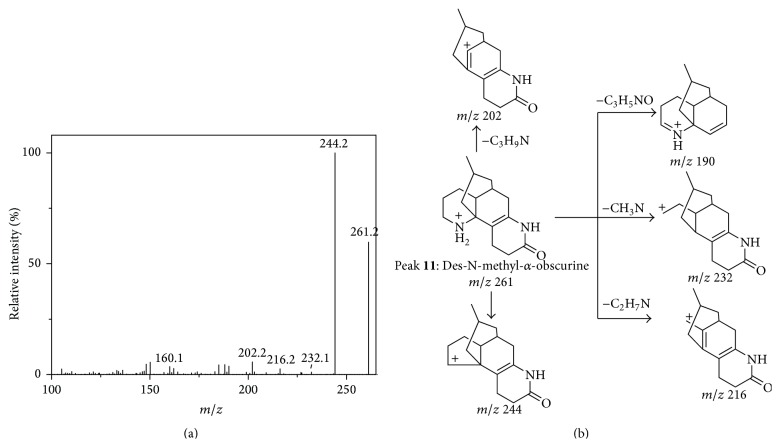
The MS/MS spectrum (a) and the proposed fragment pathways (b) of peak** 11**.

With a [M + H]^+^ ion at *m*/*z* 243 (C_15_H_18_N_2_O), peak** 14** showed the abundant fragment ion at *m*/*z* 226 and 184, which was produced by the loss of NH_3_ (17 Da) and C_3_H_9_N (59 Da), respectively. The further cleavage of ring A from fragment at *m*/*z* 226 led to the fragments at *m*/*z* 211 and 197 by the loss of NH (15 Da) and CH_3_N (29 Da), respectively. Thus, peak** 14** could be identified as lycodine [11]. Peak** 5** exhibited a [M + H]^+^ ion at *m*/*z* 273, and its fragments at *m*/*z* 230 [M + H-C_2_H_5_N]^+^ and 216 [M + H-C_3_H_7_N]^+^ could be ascribed to the loss of N and adjacent atoms. However, the fragment at *m*/*z* 256 indicated the compound to be *β*-obscurine but not obscurinine [22], because the loss of NH_3_ from obscurinine is very difficult due to the rigid structure of the latter. For peak** 11**, the intensive fragments were observed at *m*/*z* 244 [M + H-NH_3_]^+^, 216 [M + H-C_2_H_7_N]^+^, and 202 [M + H-C_2_H_7_N-CO]^+^. With the MS/MS spectrum and the proposed fragment pathway, shown in Figures 3(a) and 3(b), respectively, Peak** 11** was identified as Des-N-methyl-*α*-obscurine [3].

#### 3.2.3. Fawcettimine Type Alkaloids

Three peaks (peaks** 9**,** 12,** and** 31**) were identified and classified into fawcettimine type of alkaloids. For peak** 9**, most intensive fragment ion at *m*/*z* 230 were produced by the loss of H_2_O from its [M + H]^+^ ion at *m*/*z* 248, and peak** 9** was identified as lycoposerramine Q by comparing its MS/MS with the data reported [25]. The similar fragment ions at *m*/*z* 260, 242, 232, and 201 were observed in the MS/MS spectra of peaks** 12** and** 31**. Since peaks** 12** and** 31** exhibited the most intensive fragment at *m*/*z* 260 and 232, they were identified as palhinine B and palhinine A, according to their [M + H]^+^ at *m*/*z* 278 and 292, respectively [1].

#### 3.2.4. Miscellaneous Type Alkaloids

Major miscellaneous type alkaloids were the LAs which could not be classified into the other three types as discussed above. Based on the MS/MS analysis, peaks** 2**,** 4**,** 10**,** 15**,** 18**,** 23**,** 29**,** 30,** and** 34** were identified and classified into miscellaneous type alkaloids.

**Figure 4:**
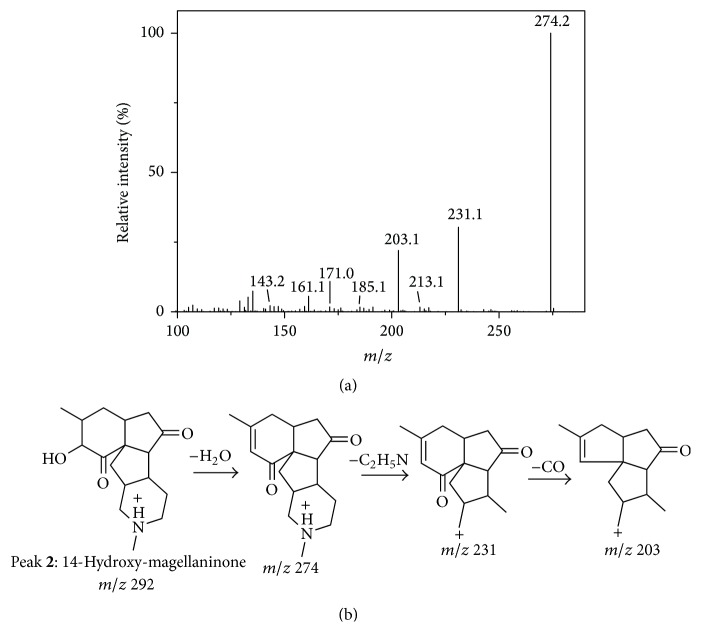
The MS/MS spectrum (a) and the proposed fragment pathways (b) of peak** 2**.

Peak** 10** showed the [M + H]^+^ ion at *m*/*z* 274 and the most abundant fragment ion at *m*/*z* 231 which was produced by the loss of its N vicinal moiety, and the further loss of CO yielded fragment ion at *m*/*z* 203. Therefore, peak** 10** was identified as magellaninone [10]. Peak** 2** exhibited the [M + H]^+^ ion at *m*/*z* 292 and the same fragments as peak** 10** with *m*/*z* at 231, 213, 203, 185, 171, 161, and 143. The same fragments of the two peaks indicated the similarity of their structures. Based on the MS/MS spectrum and proposed fragment pathway shown in Figure 4, Peak** 2** was tentatively identified as 14-hydroxy-magellaninone.

For peak** 4**, the loss of H_2_O from [M + H]^+^ at *m*/*z* 276 produced the most intensive fragment ion at *m*/*z* 258. The further loss of CO and N-vicinal moieties yielded fragment ions at *m*/*z* 230, 201, and 159. Thus, peak** 4** was identified as lycobscurine A [26]. Compared with peak** 4**, the common fragments of peaks** 18** and** 23**, at *m*/*z* 258, 230, 201, and 159, were observed. Coupled with the [M + H]^+^ at *m*/*z* 258 for peak** 18** and *m*/*z* 290 for peak** 23**, these two peaks were tentatively identified as 5,6-dehydrolycobscurine A and 5-methoxyl-lycobscurine A, respectively. As to peak** 29**, the same MS/MS spectrum with peak** 18** indicated that the two peaks could be the stereoisomers.

For peak** 30**, the product ion at *m*/*z* 218 was observed due to the loss of C_3_H_4_ from fragment at *m*/*z* 258. The loss of N vicinal moiety (C_4_H_11_N) led to the formation of product ion at *m*/*z* 203. Thus, peak** 30** with the [M + H]^+^ ion at *m*/*z* 276 was identified as magellanine [10]. For peak** 15**, the same fragment ions with peak** 30** at *m*/*z* 218 and 203 were observed. Peak** 15** was tentatively identified as Des-N-methyl-13-hydroxy-magellanine according to its [M + H]^+^ at *m*/*z* 264. The most intensive fragment ions at *m*/*z* 126 and 84 of peak** 34** were produced due to the RDA cleavage via the neutral loss (C_12_H_23_N). The loss of H_2_O yielded fragment at *m*/*z* 265; the successive loss of CH_5_N (31 Da) or CH_11_N (85 Da) led to the fragments at *m*/*z* 234 or 182. Thus, peak** 34** was tentatively identified as N-acetyl-cermizine B.

### 3.3. Comparison of LAs Compositions of* Lycopodium* from the Four Geoorigins

To illustrate the similarity of LAs of* Lycopodium* from the four origins, Similarity Evaluation System for Chromatographic Fingerprint of Traditional Chinese Medicine (Version 2004A) was employed. Compared with the average chromatogram, the similarity was 26.3%, 44.1%, 88.2%, and 93.2% for Hubei, Zhejiang, Kenya, and Guangxi, respectively. The results of similarity evaluation indicated that the distinction of soil and grown environment for* Lycopodium japonicum* may play an important role in the chemical diversity and content distinction of LAs, especially* Lycopodium japonicum* of Hubei origin.

**Figure 5:**
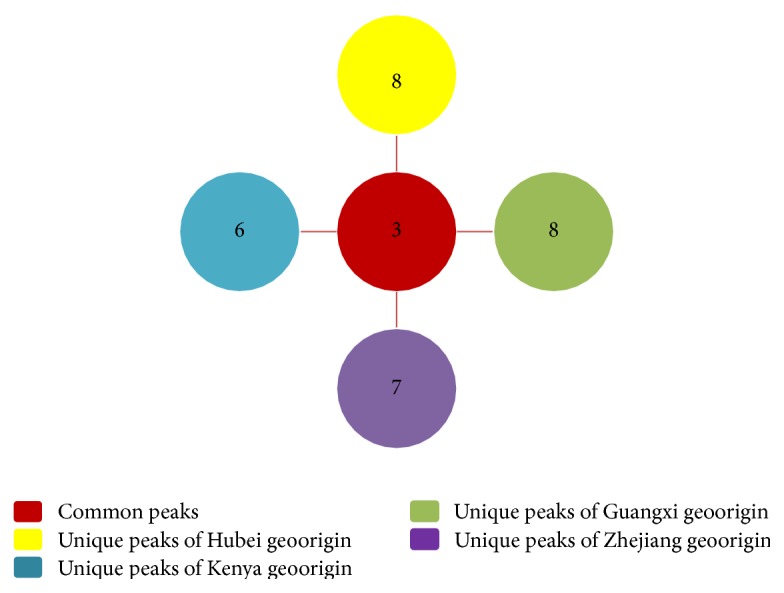
Peaks corresponding to LAs of* Lycopodium japonicum* from four geoorigins.

To further elucidate the dissimilarity of the LAs profiles, the peaks detected in* Lycopodium* japonicum from different origins were summarized. As shown in Figure 5, three peaks (**1**,** 13**, and** 26**) were the common peaks among the four samples. For unique peaks from the four geoorigins, eight peaks (**2**,** 5**,** 8**,** 20**,** 25**,** 28**,** 33**, and** 37**) were found only in sample of Hubei origin, while six peaks (**9**,** 17**,** 19**,** 36**,** 40**, and** 44**) were found only in that of Kenya origin, eight peaks (**3**,** 4**,** 7**,** 11**,** 23**,** 24**,** 31,** and** 42**) only in that of Guangxi origin, and seven peaks (**10**,** 12**,** 21**,** 35**,** 41**,** 43,** and** 46**) only in that of Zhejiang origin, respectively. In addition, some peaks were only found in two of the four origins, which are peak** 22** in Hubei and Kenya origins, peak** 27** in Hubei and Guangxi origins, and eight peaks (**6**,** 15**,** 18**,** 29**,** 30**,** 32**,** 39,** and** 45**) in Guangxi and Zhejiang origins. Moreover, four peaks (**14**,** 16**,** 34,** and** 38**) were commonly found in three geoorigin samples of Kenya, Guangxi, and Zhejiang, other than Hubei.

In most cases, the chemical differences of a medicinal plant species often resulted in pharmacological distinctions. Once phytochemical differences of plants from different geographic origins were found to be remarkable, its bioactivities might be observed distinguished. To control the quality of* Lycopodium japonicum* and its derived health products, it is of great importance to distinguish* Lycopodium japonicum* from different geoorigins. In this work, characteristic peaks corresponding to LAs which are unique for certain geoorigins with relative high intensity can be selected as markers for this purpose. Based on the fingerprinting profiles in Figure 1, peak** 2** was selected as a characteristic marker for* Lycopodium japonicum* of Hubei origin, while peaks** 9**,** 10,** and** 11** could be selected for Kenya, Zhejiang, and Guangxi, respectively.

### 3.4. Anti-HepG2 Activity of LAs

**Figure 6:**
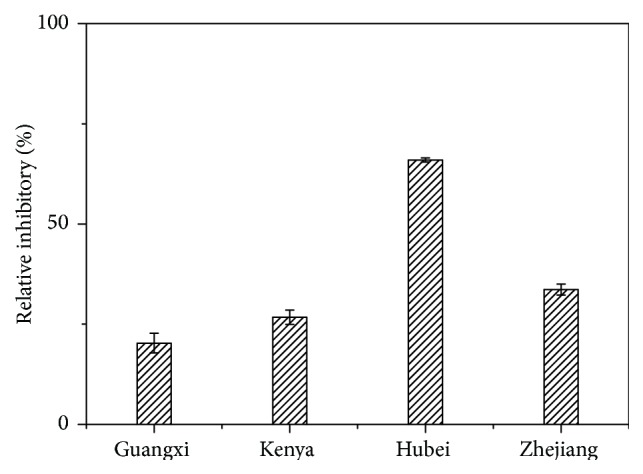
The anti-HepG2 tests on LAs from four geoorigins (the error bars indicated RSDs calculated from triplicate tests).

To explore the correlations between LAs of* Lycopodium japonicum* from four geoorigins and their respective bioactivities,* in vitro* anti-HepG2 activity tests on LAs from the four geoorigins were conducted using CCK-8 (Cell Counting Kit-8). As shown in Figure 6, total LAs of Hubei origin exhibited a much higher inhibitory rate at 65.95%, followed by Zhejiang, Kenya, and Guangxi origins at 33.62%, 26.72%, and 20.26%, respectively. In addition to the composition, the quantity of LAs from the four geoorigins may be another factor influencing the anti-HepG2 activity. The differences in the inhibitory rates of total LAs against HepG2 among the four geoorigins indicated that the soil and grown environment may contribute to the phytochemical differences of LAs and thus the discrepancies in anti-HepG2 activities. Further fingerprinting analysis implied that peaks** 2** (14-hydroxy-magellaninone) and** 28** (unidentified compound) from Hubei could be the major active components responsible for the anti-HepG2 activity.

## 4. Conclusion

In this study, HPLC-UV/ESI-MS/MS and anti-HepG2 assay were used to compare the composition and antitumor activity among the LAs extracted from Hubei, Kenya, Guangxi, and Zhejiang. To the best of our knowledge, it is the first report on the comparison of LAs from the same club moss species from four different geographic origins at the same time, suggesting that the geographic origins exerted great influence on the components. Among the identified peaks, peaks** 2**,** 9**,** 10,** and** 11** were only detected from Hubei, Kenya, Zhejiang, and Guangxi origins, respectively, and could be selected as characteristic chemical markers, which could be used for the quality control of* Lycopodium japonicum* from different geoorigins.

Further anti-HepG2 activity analysis revealed that the LAs from Hubei origin exhibited the highest inhibitory rate at 65.95% among the four geoorigins, followed by Zhejiang, Kenya, and Guangxi origins at 33.62%, 26.72%, and 20.26%, respectively. Since the chemical differences of a medicinal plant species often resulted in pharmacological distinctions, the fingerprinting analysis combined with corresponding activity assays clearly indicated that peaks** 2** (14-hydroxy-magellaninone) and** 28** (unidentified compound) from Hubei could be the major active components responsible for the anti-HepG2 activity.

To the best of our knowledge, the current research provides the most detailed phytochemical profiles of LAs in* Lycopodium japonicum* and offers valuable information for the quality control or further pharmaceutical study of this plant.

## Figures and Tables

**Table 1:** MS/MS data and the identification results of *Lycopodium* alkaloids.

Peak number	*R*_*t*_ (min)	PI-MS [M + H]^+^	MS/MS data	Identification
1	4.98	280	280, 262, 244, 236, 218, 216	Unidentified
2	6.74	292	292, 274, 231, 217, 203, 171, 161, 143	14-Hydroxy-magellaninone^dΔ^
3	8.11	280	280, 262, 244, 234, 216, 188	6*α*,8*β*-Dihydroxylycopodine^a^
4	8.92	276	276, 258, 233, 215, 201, 187, 159	Lycobscurine A^d^
5	10.31	273	273, 256, 230, 216, 188	*β*-Obscurine^b^
6	10.35	264	264, 246, 228, 218, 200, 190	Lucidioline or its stereoisomer^a^
7	10.78	280	280, 262, 244, 234, 218, 216, 188	Obscurumine A^a^
8	10.89	399	399, 381, 273, 256, 244, 230, 216	Unidentified
9	11.18	248	248, 230, 220, 213, 200, 192	Lycoposerramine Q^cΔ^
10	11.26	274	274, 256, 231, 213, 203, 185, 171, 161, 143	Magellaninone^dΔ^
11	11.37	261	261, 244, 216, 202, 190	Des-N-methyl-*α*-obscurine^b^
12	11.88	278	278, 260, 242, 232, 218, 201	Palhinine B^c^
13	13.44	294	294, 276, 262, 244, 226, 215	12-Methoxyl-lycoposerramine K^aΔ^
14	14.10	243	243, 226, 211, 197, 184	Lycodine^b^
15	14.85	264	264, 246, 228, 218, 203, 187, 173, 161	Des-N-methyl-13-hydroxy-magellanine^dΔ^
16	15.83	308	308, 248, 230, 202, 187, 171, 159, 145	*α*-Lofoline^a^
17	16.16	246	246, 228, 218, 204, 190, 176, 162, 150, 124	Anhydrolycodoline or its stereoisomer^a^
18	16.47	258	258, 230, 216, 199, 185, 157, 143	5,6-Dehydrolycobscurine A or its stereoisomer^dΔ^
19	16.57	289	289, 247, 230, 218, 190, 174, 145	5-N-Acetyl-4,5-dehydrolycopodine^aΔ^
20	16.84	566	566, 548, 453, 435, 339, 322, 228, 209, 114	Unidentified
21	17.33	278	278, 260, 242, 234, 232, 216, 192, 174	Lannotinidine J^a^
22	17.37	292	292, 274, 260, 232, 229, 217, 201	Unidentified
23	17.37	290	290, 258, 230, 227, 216, 201, 199, 185, 159, 157	5-Methoxyl-lycobscurine A^dΔ^
24	17.84	366	366, 348, 288, 228, 200, 186, 173, 159	Unidentified
25	17.95	381	381, 364, 363, 346, 338, 324, 306, 294, 278	Unidentified
26	18.22	679	679, 661, 566, 548, 452, 435, 341, 322, 228	Unidentified
27	18.90	260	260, 242, 232, 214, 186, 160	8-Alkenyl-lycoposerramine K or 6-alkenyl-gnidioidine^aΔ^
28	19.71	372	372, 354, 274, 260, 242, 232, 218, 189, 176	Unidentified
29	20.03	258	258, 230, 216, 201, 199, 185, 173, 162, 159, 157	5,6-Dehydrolycobscurine A or its stereoisomer^d^
30	21.03	276	276, 258, 245, 234, 218, 203, 190, 176	Magellanine^d^
31	21.28	292	292, 274, 260, 242 232, 201, 187, 175, 159	Palhinine A^c^
32	22.65	278	278, 260, 242, 232, 214, 160	8*β*-Hydroxylycoposerramine K^a^
33	23.33	260	260, 242, 229, 217, 211, 203, 185, 169, 161, 70	Unidentified
34	23.87	307	307, 265, 234, 217, 182, 161, 149, 135, 126, 84	N-Acetyl-cermizine B^dΔ^
35	24.41	246	246, 228, 218, 204, 190, 176, 162, 150, 124	Anhydrolycodoline or its stereoisomer^a^
36	25.62	545	545, 286, 272, 260, 243, 225, 215, 197, 183, 155	Unidentified
37	26.04	296	296, 278, 260, 234, 218, 216, 192	Miyoshianine C^a^
38	27.43	262	262, 244, 234, 226, 216, 201, 187, 175, 161	Lycoposerramine K^a^
39	28.02	531	531, 513, 298, 272, 258, 230, 216, 202, 188, 176	Unidentified
40	28.69	334	334, 302, 276, 260, 246, 233, 218, 215, 191, 187	Unidentified
41	29.63	545	545, 286, 272, 243, 215, 197, 183, 155	Unidentified
42	31.01	579	579, 334, 290, 272, 246 (247), 187	Unidentified
43	32.85	304	304, 286, 276, 262, 258, 244, 277, 218, 201, 189	Unidentified
44	33.27	519	519, 274, 258, 243, 231, 205, 185, 171	Unidentified
45	34.16	390	390, 372, 236, 328, 285, 229, 218, 200, 144	Unidentified
46	35.06	304	304, 286, 276, 262, 258, 243, 230, 210, 201	Unidentified

^Δ^Firstly reported in *Lycopodium japonicum* species.

^a^Lycopodine type, ^b^lycodine type, ^c^fawcettimine type, and ^d^miscellaneous type.
